# Meal or mate: Exploring the evidence of sexual cannibalism among amphibians

**DOI:** 10.1002/ece3.11576

**Published:** 2024-06-12

**Authors:** John Gould, Chad T. Beranek

**Affiliations:** ^1^ School of Environmental and Life Sciences University of Newcastle Callaghan New South Wales Australia; ^2^ FAUNA Research Alliance Kahibah New South Wales Australia

**Keywords:** conspecific interaction, intra‐specific predation, *Litoria aurea*, sexual conflict, sexual dimorphism

## Abstract

Active forms of cannibalism that involve predation of live conspecifics occur widely among amphibians, most notably by tadpoles that feed on each other and adults that feed on juveniles. In contrast, cannibalism among amphibian adults (adult–adult cannibalism) is less often reported and there have been no investigations on the occurrence of sexual cannibalism in this group to date. In this study, we present an observation of potential sexual cannibalism involving an adult female green and golden bell frog, *Litoria aurea*, preying on a conspecific adult male during the species' breeding season. By comparing our observation to the available literature, we show that adult–adult cannibalism among amphibians is rare but tends to be committed by females against their male counterparts. We thus suggest that the occurrence of sexual cannibalism should be extended to include this group, where sexual size dimorphism occurs widely among adults that congregate spatially during breeding periods, both predictors of intra‐specific predation. We hypothesise that amphibian females may be able to exploit male advertisement calls to differentiate suitable partners from potential prey and that male individuals are vulnerable to sexual cannibalism as they must risk attracting and physically exposing themselves to females in order to reproduce. Our findings reveal the complex dynamics that exist within adult amphibian populations, suggesting that females may have a choice when deciding how to interact with and utilise their male counterparts. As our findings are preliminary, based on a small sample size of records, including several from captive individuals, we encourage authors to publish their observations of cannibalism in the field, including unsuccessful attempts, to confirm the presence of sexual cannibalism in this group.

## Graphical abstract

Sexual cannibalism is a form of conspecific predation among adults, typically committed by females that consume potential or actual mating partners. We provide an observation of cannibalism involving an adult female green and golden bell frog, *Litoria aurea*, preying on a conspecific adult male during the species' breeding season. By comparing our observation to the available literature, we show that adult–adult cannibalism among amphibians is rare but tends to be committed by females against their male counterparts and propose that amphibians possess traits that may promote the evolution of sexual cannibalism.

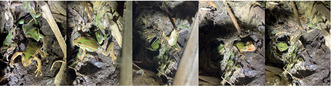

## 1 INTRODUCTION

Cannibalism can involve active forms of intra‐specific predation whereby individuals' prey on live conspecifics (Heinen & Abdella, 2005), which contrasts with the passive consumption (i.e. scavenging) of conspecific detritus/carrion. This is generally an opportunistic foraging strategy and occurs widely across animal groups (Fox, 1975; Polis, 1981). Adaptive benefits at the individual level include reductions in resource competition and provision of high‐quality nutrients that lead to growth and development advantages (Meffe & Crump, 1987; Wildy et al., 1998), particularly during periods of food shortages (Amstrup et al., 2006). These benefits may be offset by associated costs including the risk of injury, disease transmission and reduced fitness by the killing of relatives (Crump, 1992; Fox, 1975; Pfennig, 1997; Williams & Hernández, 2006). At the population level, cannibalism may contribute to the regulation of population dynamics, including recruitment, population size and age structure (Fox, 1975; Hopkins et al., 2023; Polis, 1981; Whiteman & Wissinger, 2005) as well as dispersal (Rudolf et al., 2010). Identifying the presence and extent of cannibalism within populations may reveal complex interactions that exists between conspecifics that have been overlooked.

Among amphibians, active forms of cannibalism have been widely documented (Crump, 1992; Measey et al., 2015; Polis & Myers, 1985; Toledo et al., 2007), primarily involving adult life stages preying on juveniles, which have limited dispersal capacity, and unhatched embryos, which are immobile. This could be a strategy used by adults to reduce future competition or predation of their own offspring as younger conspecifics transition from being prey to a predator with increasing body size (i.e., status inversion; Kaplan & Sherman, 1980; Toledo et al., 2007). Cannibalism is also commonly exhibited by tadpoles that prey on conspecifics at the same life stage or unhatched embryos, including siblings (filial cannibalism) (Dugas et al., 2016; Gould et al., 2022). This may provide tadpoles fitness benefits by reducing competition for resources and increasing developmental rate, the latter being especially important for species that occupy ephemeral habitats where tadpole cannibalism is more commonly exhibited. Cannibalism is associated with situations where individuals are exposed to high population densities, low food resources and high levels of resource sharing (Fox, 1975; Polis, 1981). Among amphibians, this occurs under two main scenarios: when embryos hatch and large tadpole populations are rapidly exposed to limited resources and space (Gould et al., 2022) and when recently metamorphed juveniles make a transition from aquatic to terrestrial habitats that are used by adults (Measey et al., 2015). Yet, other concentrations of conspecifics also occur among amphibians, most notably the movement of reproductively active adults to breeding sites, including the formation of large and dense adult aggregations (e.g. Roberts, 1994), which should also provide suitable conditions for cannibalism to occur.

Yet, forms of cannibalism that involve adults preying on each other appear to be rare among amphibians, as noted by Muñoz Saravia et al. (2020). Indeed, in their review, Polis and Myers (1985) showed that adult–adult cannibalism accounted for few records in the literature, mirroring the low rate of its occurrence among other vertebrate groups including reptiles and mammals (Polis et al., 1984; Polis & Myers, 1985). This could be due to the negative effects of exploiting larger conspecifics as prey, including excessive handling times, reduced rate of success, reduced mobility upon digestion and increased risk of injury or death (Kaiser et al., 2016; Measey et al., 2015; Muñoz Saravia et al., 2020), despite the benefits of relatively higher nutrient intake.

Out of all forms of cannibalism, sexual cannibalism has received limited attention among amphibians (Elgar, 1992; Elgar & Schneider, 2004), despite species in this group commonly displaying sexual dimorphism and size disparity being a key predictor of intra‐specific predation (Measey et al., 2015; Polis, 1981; Shine, 1979). During sexual cannibalism, adult individuals, typically females (but see Glaudas & Fuento, 2022), consume potential or actual mating partners; the most well‐known examples being among predatory invertebrates (Elgar, 1992). Sexual cannibalism is considered a form of sexual conflict and may provide fitness benefits for the participating sex but also for preyed upon sex dependent on the timing of consumption relative to copulation (Zuk, 2016). For the participating sex, benefits include obtaining nutrients that can increase fecundity (adaptive foraging; Barry et al., 2008; Newman & Elgar, 1991) and preventing copulation with non‐preferred mates (mate choice; Mark A Elgar & Nash, 1988; Persons & Uetz, 2005). For the preyed upon sex, it may increase fitness if consumption occurs after successful copulation, as the nutritional value provided to the mating partner may increase the chance of their offspring surviving (Buskirk et al., 1984). Unlike other forms of cannibalism, there is a relatively low risk of individuals who participate in sexual cannibalism killing close relatives. However, there may be negative population‐level effects including reductions in population viability and extinction, particularly in the face of environmental change (Fisher et al., 2018), via the removal of available mates (Hurd et al., 1994). The potential for sexual cannibalism to occur among amphibians requires further investigation, including the conditions that may promote its occurrence and the consequences on adult population dynamics.

In this study, we present an observation of potential sexual cannibalism involving an adult female green and golden bell frog, *Litoria aurea*, preying on a conspecific adult male during the breeding season. We have subsequently reviewed the literature to explore the occurrence of adult–adult cannibalism among amphibians, including sexual cannibalism, and the conditions that may favour its occurrence in this group. For completeness, we test if there is a size‐based sexual dimorphism present in *L. aurea* and if females were larger than males that they consumed when reported in the literature, which tests a key prediction of sexual cannibalism.

## 2 METHODS

### 2.1 Fieldwork

Nocturnal visual encounter surveys were conducted on Kooragang Island, NSW, Australia (32.86109° S, 151.73294° E) from September to April over three consecutive breeding seasons (2021–2023) to monitor one of the largest extant *L. aurea* populations within its natural distribution (Pyke & White, 2001). Approximately 90 freshwater ponds ranging from natural and semi‐natural to artificial and constructed were surveyed across the island to detect *L. aurea* individuals. Each pond survey was completed by two or more researchers wearing head torches by wading through the water for a total of 30 survey minutes, primarily targeting riparian emergent vegetation and the adjacent terrestrial landscape up to 10 m from the water. Additionally, temporary exclusion fencing installed around a wetland complex undergoing remediation in the southern portion of the island was surveyed during the 2021 and 2022 breeding seasons. Fence surveys were conducted by walking the entire perimeter of the fenceline, in order to collect and translocate frogs to nearby wetlands for their protection (see Gould et al., 2024, for more details).

Individuals were collected when possible and snout–vent length (SVL) were measured using dial callipers (General Tools, Seacaucus, NJ, USA) to the nearest 0.1 mm. The sex of adult individuals was determined based on animal size and the presence/absence of nuptial pads as per Gould et al. (2021), with individuals larger than 58 mm SVL without nuptial pads defined as adult females and individuals of any size with nuptial pads defined as adult males; this size threshold for females has been set based on the smallest females found to be gravid in this population (Colin McHenry, personal communication). Sex was also determined based on sexual dimorphic colouration; males tend to have yellowing of the vocal sac, whereas females have an opaque white colouring across their ventral surfaces (Gould & McHenry, 2024). Individuals were chipped with a uniquely coded passive integrated transponder upon initial capture for identification purposes. Previously captured and chipped animals were scanned with a Trovan radio frequency identification reader (Microchips Australia Pty Ltd, Keysborough, Vic) to record identity codes. All frogs were subsequently released to their point of capture.

Across all surveys, predatory behaviours exhibited by *L. aurea* individuals were recorded without interference using an iPhone 12 (Apple Corporation, Cupertino, CA, USA). These individuals were not collected, to prevent disturbing them with captured prey or because the process of recording without interference provided them an opportunity to escape. Sex and staging for individuals engaging in such behaviours were thus determined visually.

We used linear mixed modelling to determine if there was a difference in the SVL of adult males and females, with SVL included as the response variable and sex (male and female) included as a categorical explanatory variable. On some occasions, individuals were captured several times within a given breeding season. We thus averaged all within‐season SVL measurements to ensure only a single value was included for all individuals within each yearly cohort. Multiple values of the same individual were only included if they were captured in more than one breeding season. To account for repeat measures of individuals between years, we included animal ID as a random variable. Additionally, we also included year as a random variable to account for differences in the adult cohort between seasons. As our model included only one explanatory variable that was categorical, we analysed model results using ANOVA. All statistical analyses were performed in R version 4.0.4 (R Team, 2020) using the Lme4 package (Bates et al., 2015). Additionally, the extent of sexual size dimorphism present within the population was calculated using the size dimorphism index (SDI = mean body size_females_/mean body size_males_ – 1) (Lovich & Gibbons, 1992).

### 2.2 Literature search

We conducted a literature search of records of adult–adult cannibalism among amphibians between 20 December 2023 and 15 January 2024. Literature was found on Google Scholar using key search terms ‘cannibalism’ OR ‘predation’ with ‘And (amphibian OR salamander OR newt OR anuran OR frog OR toad)’, while excluding the words ‘tadpole’, ‘egg’, ‘embryo’, and ‘juvenile’ within study titles. We only searched for publications in English. The title of each paper was manually assessed for relevancy based on whether they were concerned with cannibalism specifically involving adult amphibians. We also checked the reference lists of studies deemed relevant. When cases of adult–adult cannibalism were found, we recorded the species, sex/stage and SVL of predator and prey individuals, whether prey was consumed entirely or partially (i.e. body regions still outside of the mouth) or escaped, and the manner of consumption (head or vent first), as well as the setting (wild versus laboratory), location and year of the observation, and whether the observation occurred within the species' breeding season. Photographs present in these studies were used to determine relative size differences between predator and prey individuals if size measurements were not provided. These data were used to test whether the size of conspecific prey tended to be smaller than the size of predators using a one‐tailed t‐test. To account for differences in the sizes of amphibian species, we standardised each prey size to be a percentage of the size of their predator, with all predator sizes standardised to 100.

## 3 RESULTS

### 3.1 Sexual size dimorphism in *Litoria aurea*

**FIGURE 1:**
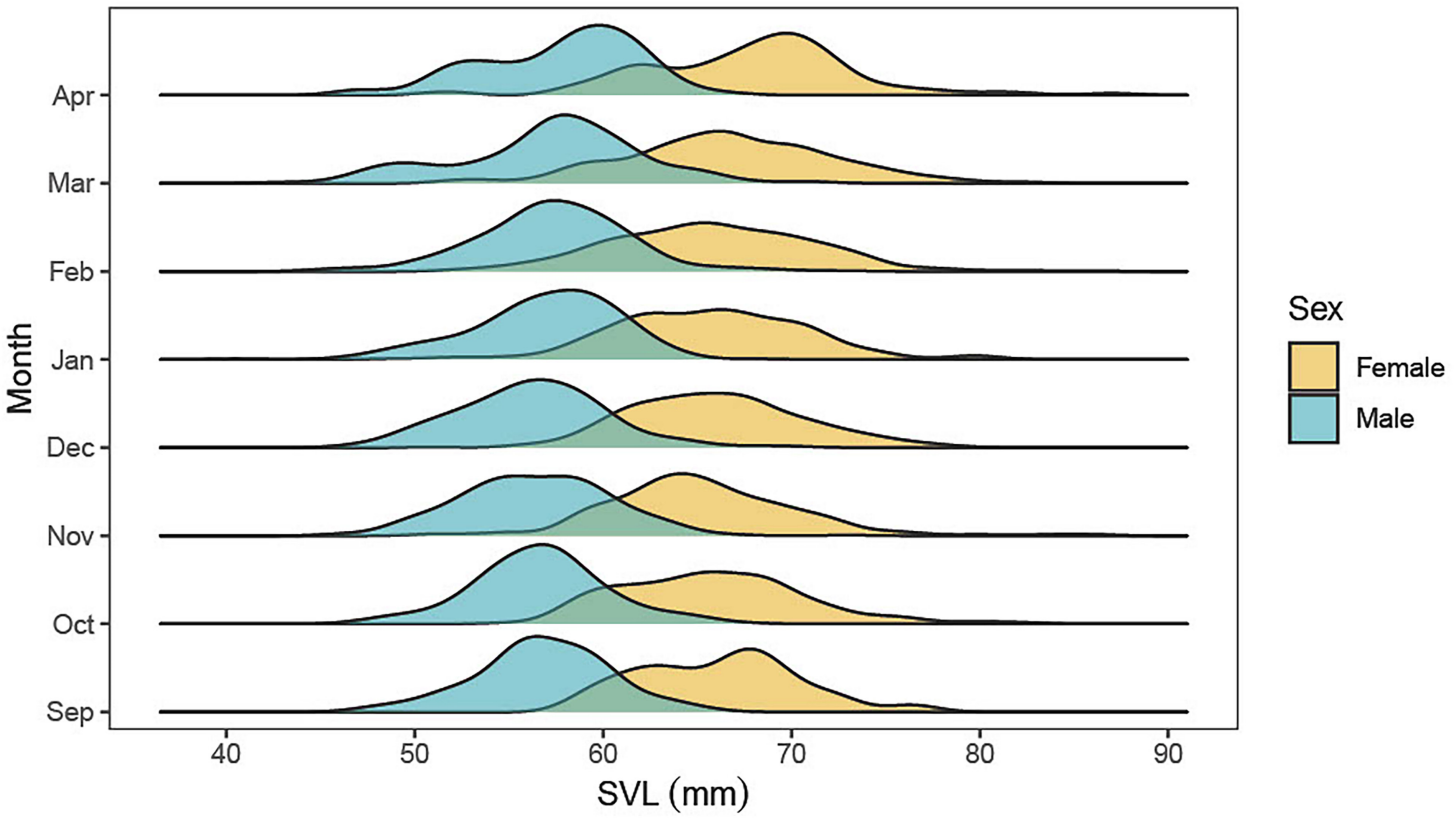
Body size comparison of *Litoria aurea* adults across the breeding season. Frequency distribution of snout–vent length (mm) of females (orange) and males (blue) from Kooragang Island, NSW, Australia, separated by month (September to April) of capture. Data for three consecutive breeding seasons, 2021–2023, have been pooled for each month.

A total of 2532 unique adult *L. aurea* individuals were captured and sexed across the three consecutive breeding seasons, including 1066 females and 1466 males. Sexual size dimorphism was detected (SDI = 16%), with female SVL ranging between 49 and 87 mm (mean = 66 mm, SD = 5) and male SVL ranging between 43 and 73 mm (mean = 56 mm, SD = 4) (Figure 1; ANOVA: *F*_(1,2450)_ = 2495, *p* < .0001). We captured two amplecting pairs during the study period, with the size difference between male and female partners being 10.7 mm (male = 57 mm, female = 68 mm) and 1 mm (male = 73 mm, female = 74 mm).

### 3.2 Heterospecific predation by *Litoria aurea*

**FIGURE 2:**
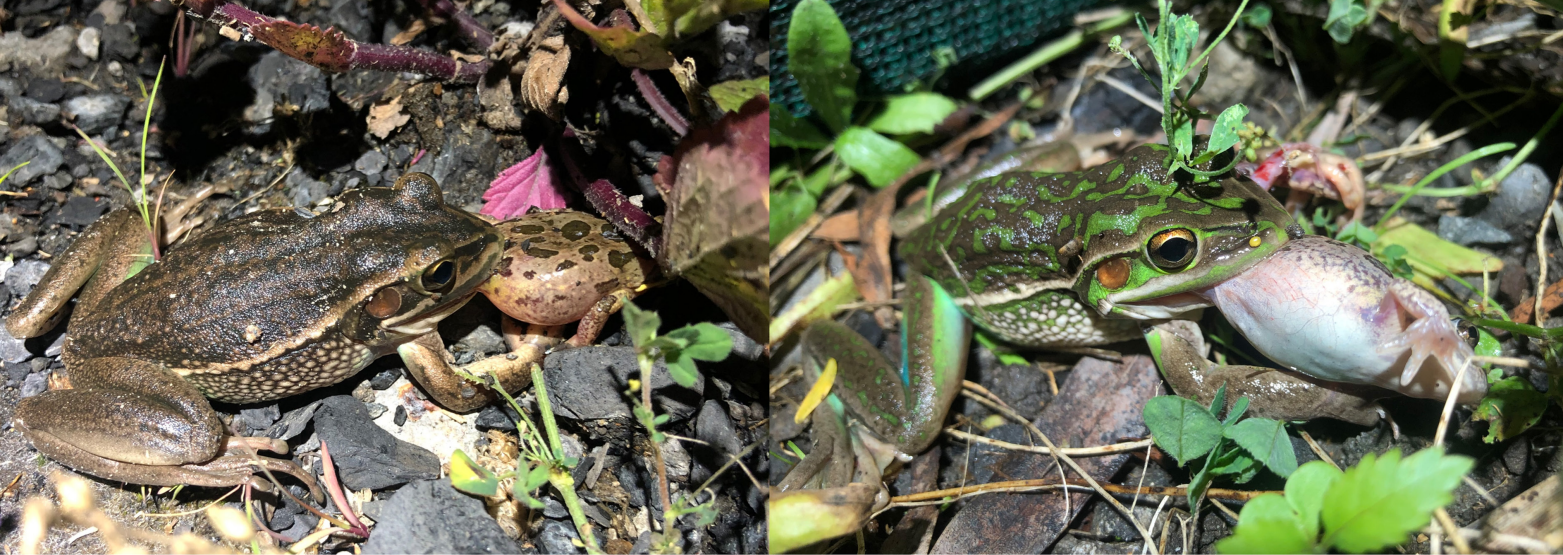
Adult female green and golden bell frogs, *Litoria aurea*, preying on adult spotted marsh frogs, *Limnodynastes tasmaniensis* in proximity to an exclusion fence on Kooragang Island, NSW, Australia. The females can be seen swallowing their frog prey vent first, with the back legs already consumed.

During the 2021 breeding season, we made two observations of *L. aurea* consuming other frog species in proximity to exclusion fencing in the southern portion of Kooragang Island (Figure 2). In both cases, an adult female was observed on the ground with a spotted marsh frog, *Limnodynastes tasmaniensis*, partially consumed but still alive. Both prey frogs were adults based on size, with the sex of one unknown and the other likely female based on the presence of flattened phalanges. The *L. tasmaniensis* individuals were being swallowed from the rear (vent first), with the legs already ingested and the heads still exposed. The prey individuals also showed inflation of their lungs, possibly a defensive mechanism to resist being consumed (Kaiser et al., 2016). It was unconfirmed whether these predation attempts were successful.

**FIGURE 3:**
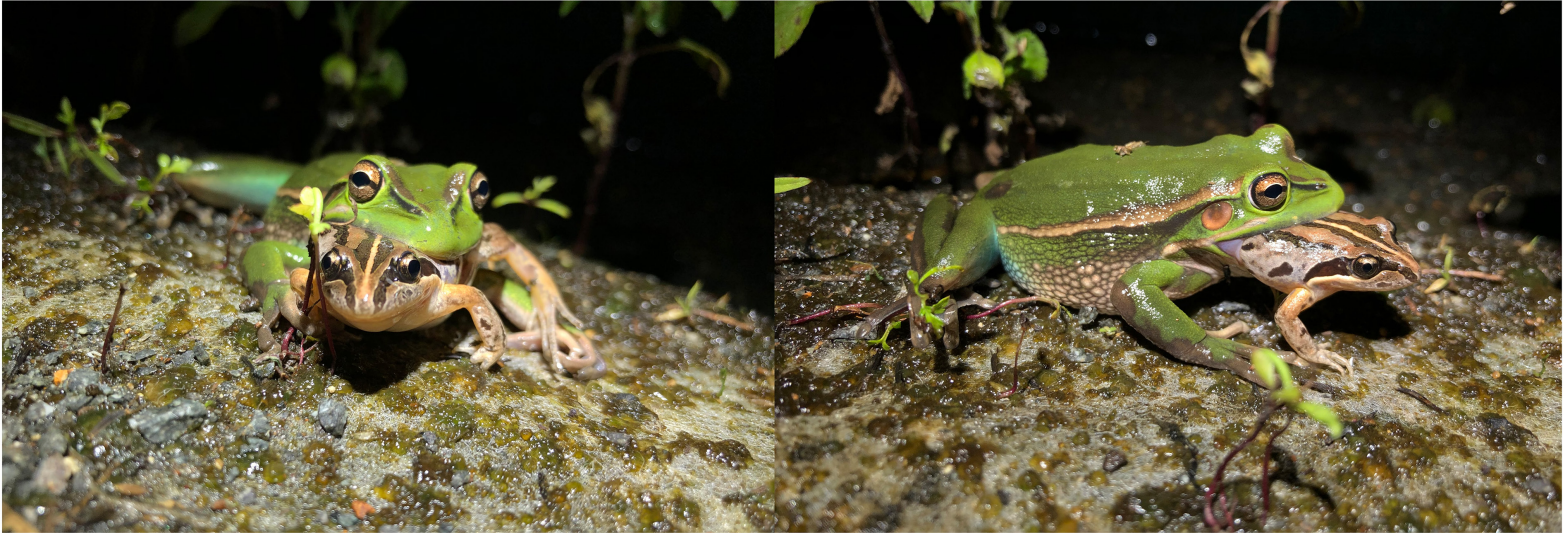
Front and side view of an adult female green and golden bell frog, *Litoria aurea*, preying on a juvenile striped marsh frog, *Limnodynastes peronii*. The frog prey has been swallowed from the rear up to the middle of the body.

In the same breeding season, we also made a similar observation where an *L. aurea* female was found on the ground preying on a striped marsh frog, *Limnodynastes peronii*, at the edge of a permanently constructed pond approximately 200 cm from the water's edge (Figure 3). The *L. peronii* individual was likely a juvenile based on its size, was still alive and had been partially consumed vent first, with the front legs and head exposed. The prey frog could be seen trying to escape capture by movement of the front limbs, but the female held on throughout the observational period. It was unconfirmed whether this predation attempt was successful.

### 3.3 Adult–adult cannibalism in *Litoria aurea*

**FIGURE 4:**
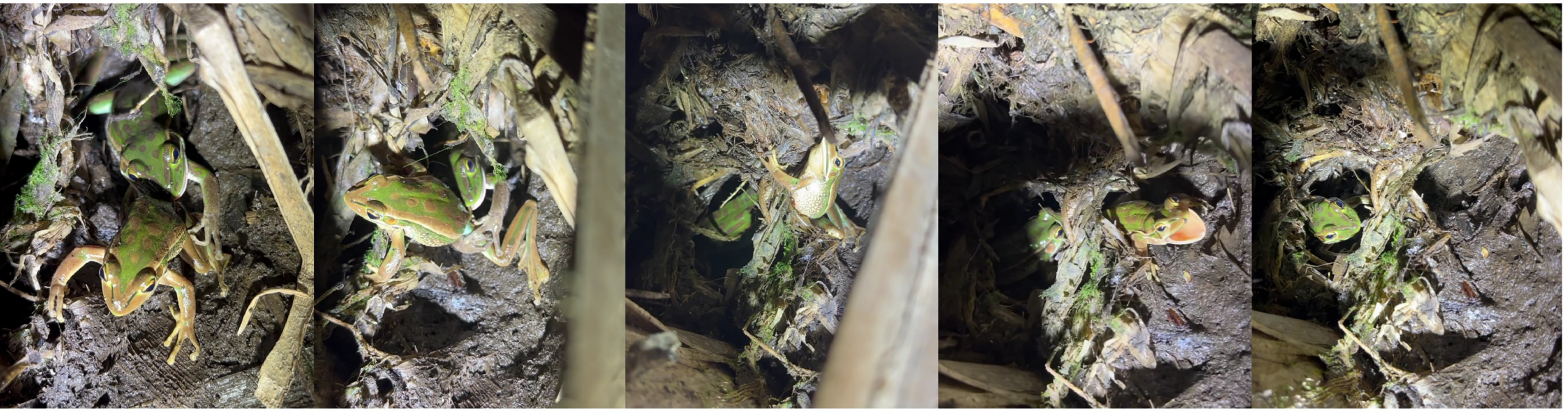
Time series showing an adult female green and golden bell frog, *Litoria aurea*, cannibalising an adult male at the edge of a permanent wetland pond on Kooragang Island, NSW, Australia. The female can be seen gripping the right hind limb of the male and dragging it down into a natural hole formation before the male escapes.

We observed an adult female *L. aurea* in a display of predatory activity, cannibalising an adult male in December 2023 (Figure 4, Video 1). The pair was found 150 cm from the water's edge of a permanent, natural pond that had partially dried, near a sparse stand of Cumbungi reeds, *Typha orientalis*. The female was situated at the entrance of a natural hole formation in the bank of the pond and had its mouth around the top of the male's right thigh, which had been entirely ingested. Over a period of 50 s, the female maintained its grip on the male and dragged it down into the hole. The captive male emitted several distress vocalisations and tried to resist being pulled further into the hole by gripping onto nearby vegetation. The male eventually escaped the female's grip and rapidly moved out of the hole. We did not detect any other forms of cannibalism, including those involving other life stages.

**VIDEO 1:** Video recording of an adult *Litoria aurea* female cannibalising a conspecific adult male at the edge of a permanent wetland pond on Kooragang Island, NSW, Australia.

### 3.4 Literature review

**FIGURE 5:**
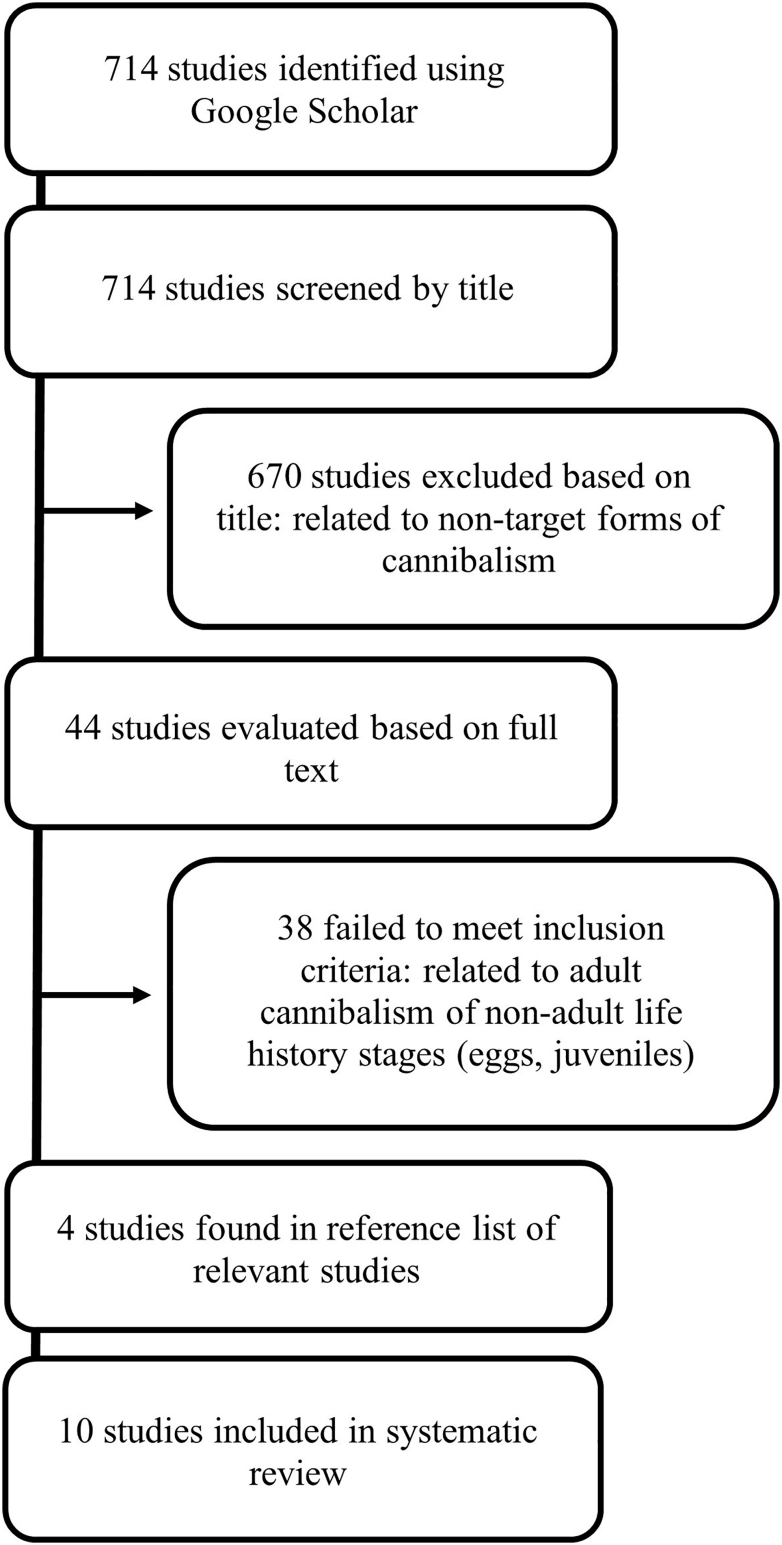
Flow chart indicating the steps undertaken during the systematic review process, including literature search, study exclusion and study evaluation.

Our search terms identified 714 studies, with 670 excluded based on an evaluation of their titles, and a further 38 excluded after an evaluation of the full text (Figure 5). After the inclusion of studies found in the reference lists, we obtained a total of 15 records of adult‐adult cannibalism among 10 amphibian species, including nine observations obtained from the wild and six from captive individuals (Table 1). The cannibal was an adult female in 9 out of the 15 cases (60%), which jumped to 9 out of 11 cases (82%) when those involving adults of unknown sex were removed. The prey of the adult females were mostly males (45%), followed by other females (33%), and adults of unknown sex (22%). Across all records, most instances of cannibalism (80%) occurred within the respective species' breeding season. On average, conspecific prey was 37% smaller (SD = 15, range = 4% to 52%) than their predators (*t* = 7.98, df = 9, *p* < .0001).

**TABLE 1:** Records of adult–adult cannibalism among amphibians in the literature.

Species	Predator sex	Prey sex	Predator SVL (mm)	Prey SVL (mm)	Size difference (%)	Consumption	Endpoint	Year	Location	Within breeding season?	Authors
*Osteopilus septentrionalis*	Female	Male	100^a^	62^a^	38	Head first	Escaped	2011	Wild	Yes^b^	Kaiser et al. (2016)
*Telmatobius culeus*	Female	Male	119	64	47	Head first	Consumed	2015	Captive	Yes^b^	Muñoz Saravia et al. (2020)
*Pseudis minuta*	Female	Male	44	23	48	Head first	Consumed	2009	Wild	Yes	Lombardo et al. (2010)
*Telmatobius culeus*	Female	Male	103	49	52	Head first	Consumed	2011	Wild	Yes^b^	Muñoz Saravia et al. (2020)
*Eurycea rathbuni*	Female	Female	47	45	4	Vent first	Partially consumed	2022	Captive	Yes^b^	Dobbins et al. (2023)
*Osteopilus septentrionalis*	Female	Female	81	43	47	Vent first	Escaped	2016	Wild	Yes^b^	Kaiser et al. (2016)
*Telmatobius culeus*	Female	Female	119	62	48	?	?	2016	Captive	Yes^b^	Muñoz Saravia et al. (2020)
*Telmatobius culeus*	Female	Unknown	135	‐	‐	?	?	2013	Wild	Yes^b^	Muñoz Saravia et al. (2020)
*Lepidobatrachus llanensis*	Female	Unknown	93	80	14	?	Consumed	1974–75	Wild	Yes	Hulse (1978)
*Telmatobius culeus*	Male	Female	91	57	37	Vent first	Partially consumed	2017	Captive	Yes^b^	Muñoz Saravia et al. (2020)
*Gastrotheca cornuta*	Male	Male	‐	‐	‐	Head first	Partially consumed	2005–2010	Captive	?	Gagliardo et al. (2010)
*Euphlyctis cyanophlyctis*	Unknown	Unknown	150	117	22	Head first	Consumed	2013	Captive	No	Rao and Shukla (2014)
*Andrias japonicus*	Unknown	Unknown	505	345	32	Head first	Consumed	2019	Wild	No	Nakagawa (2020)
*Ceratophrys pierot*	Unknown	Unknown	‐	‐	‐	?	?	1951	Wild	Yes	Cei (1955)
*Indirana leithii*	Unknown	Unknown	‐	‐	‐	Vent first	Escaped	2019	Wild	Yes	Kulkarni et al. (2020)

*Note*: The sex and size (snout–vent length; SVL) of adult cannibals and their conspecific adult prey have been provided, along with the direction of prey consumption (heard first or vent first), the endpoint of the record (whether the prey escaped, was entirely consumed or only had some body regions engulfed by the mouth of their predator), the year and location of each record, and whether the record occurred in the species' breeding season.

^a^ SVL values are relative between predator and prey and not true sizes.

^b^ Species does not have a defined breeding season but may have peak breeding months or periods.

## 4 DISCUSSION

We show that *L. aurea* adults feed on heterospecific amphibians and are cannibalistic towards conspecifics adults (adult‐adult cannibalism). The latter is a relatively rare form of intraspecific predation among amphibians compared to those involving conspecifics at different life stages (e.g. adult–juvenile cannibalism) or earlier life stages (e.g. tadpole–tadpole cannibalism). This is despite adult amphibians being generalist feeders (polyphagous) and often gathering in high densities (Toft 1985; Wells, 2007), both of which strongly correlate with the presence of cannibalism among animals (Maritz et al. 2019). Additionally, our observations in the field and review of the literature provide evidence of sexual cannibalism among amphibians that is perpetrated by adult females during breeding periods, fitting the general pattern in other animal groups (Barry et al., 2008; Elgar, 1992). Our findings reveal the complex dynamics that exist within adult amphibian populations and particularly between the sexes, suggesting that females may have a choice when deciding how to interact with and utilise their adult male counterparts.

We found few instances of adult‐adult amphibian cannibalism reported in the literature, which could be a result of the rarity of its detection and/or occurrence. Indeed, we only observed one instance of adult–adult cannibalism out of more than 2000 *L. aurea* observations during our monitoring of the Kooragang Island population. While this would suggest that such form of cannibalism is a rare occurrence, we also rarely observed more typical interactions between adults during the breeding season such as amplexus and rarely observe predation in general (Beranek et al., 2023), highlighting the issue of detectability of behaviours that are relatively short‐lived. Similarly, as cannibalism tends to occur in species with general and opportunistic feeding habits, it is expected that conspecifics make up a small proportion of their dietary intake in terms of frequency, which could result in low detectability and thus reporting of its occurrence in the wild (Glaudas & Fuento, 2022). Another possibility is that preying on and eating conspecific adults is much more difficult and less successful compared to exploiting earlier life stages that are smaller, which could reduce its occurrence and/or detection if escape is more common. For example, the *L. aurea* male preyed upon in our own observation was able to escape from its female predator, and unsuccessful predation attempts by adults have also been recorded in some species (e.g. Kaiser et al., 2016) but not others (e.g. Muñoz Saravia et al., 2020). If escape has not occurred, dietary analysis may reveal instances of sexual cannibalism after consumption has already occurred and visual detection is no longer possible.

Amphibians possess a variety of traits that may increase the chance of sexual cannibalism occurring. In particular, adult amphibians congregate during breeding periods to share similar habitats spatially and temporally, increasing the chance of encounters and making it easier for predators to find their prey (Measey et al., 2015). Amphibians also show increased physical contact with each other during the breeding period (e.g. amplexus, male–male fighting; Greene & Funk, 2009), increasing opportunities for predation to occur as individuals are already in close proximity to complete other behaviours. Along this line of logic, explosive breeding species, which congregate in large breeding masses and choruses (Wells, 2007), may be more likely to engage in sexual cannibalism. Many amphibians are also sexually dimorphic in size (Shine, 1979), with females tending to be larger than males and thus having an advantage that may afford them a greater ability to exploit the opposite sex as a potential food resource (Muñoz Saravia et al., 2020). The occurrence of asymmetric predatory interactions between the sexes is likely to be dependent on the severity of their size difference, which influences disparities in both speed and strength that would be expected as prerequisites to enable one sex to physically dominate over the other for prey capture to be possible without the assistance of other strategies such as envenomation. For *L. aurea*, the average size difference between adult females and males on Kooragang Island is approximately 16%, which appears to be sufficient to allow females to exert predation pressure on their male counterparts. It also appears the *L. aurea* females are more likely to exert the same pressure on sympatric amphibians.

Greater sexual dimorphism may also increase the ability of one sex to consume another by reducing post‐capture constraints related to prey size. This may be particularly critical for amphibians, which swallow their prey alive and whole and are thus gape‐limited, restricting maximum prey sizes to individuals that can fit within the mouth (oral gape). This contrasts with sexually cannibalistic invertebrates that consume their prey in pieces (e.g. Barry et al., 2008), where there may not be any benefit of sexual dimorphism in terms of improving prey processing and consumption. Our field observations indicate that adult female *L. aurea* have the capacity to consume adult heterospecific amphibians whole, such as *L. tasmaniensis*, which have an adult SVL of 42 mm and 47 mm for males and females, respectively (Barker et al., 1995), approximately 60%–70% the average size of adult *aurea* females on Kooraganvg Island. This is within the size range of adult male *L. aurea* we have detected in the island population, providing further evidence that some males are sufficiently small enough for sexual cannibalism by females to be physically possible in this species.

There are two possible scenarios that lead to sexual cannibalism among amphibians: (1) females that have moved towards sites of male congregation, selected a mate and deposited their eggs exploit the opportunity to prey on nearby conspecifics with little additional investment in travel, with the release of eggs providing the necessary space within the body cavity to consume relatively large prey or (2) females of all reproductive statuses move towards and exploit male congregations to prey on conspecifics. As the males of most amphibians emit calls during the breeding period to attract a mate (Duellman & Trueb, 1986), they may be a relatively easy prey type for conspecific females to detect as their auditory system is tuned to be sensitive to the call of these would‐be suitors to improve mate recognition and acquisition (matched filter hypothesis; Capranica & Moffat, 1983; Moreno‐Gómez et al., 2013). A unique situation thus arises where it is the prey that is emitting a cue that makes them vulnerable to detection by conspecific female predators, under the impression it is instead attracting these individuals as potential breeding partners. This makes it difficult for males to conceal their presence or reduce detectability from such a predator type, as they must risk predation being the consequence of encountering a female for the chance to reproduce with her.

It seems counterintuitive for female amphibians to consume available mates if it reduces their chance to reproduce. However, female amphibians are generally the limiting sex due to strong sex biases towards males at breeding sites (Richards & Alford, 2005; Vasconcellos & Colli, 2009), offering them the ability to be choosy. This is apparent for *L. aurea*, where there are typically more males congregated at breeding ponds that remain present for extended periods, resulting in a highly male‐biased sex ratio (Beranek et al., 2022), and fewer females that leave the congregation after they have deposited their eggs. Additionally, the exploitation of adult males as a food resource would incur significant benefits for females if successful, such as provisioning them a prey of high nutritive value and supporting their increased nutritional needs during the breeding period associated with egg production (Muñoz Saravia et al., 2020).

If female amphibians can determine the quality of nearby suitors based on their call attributes (Searcy & Andersson, 1986), this trait may be used to differentiate males and dictate whether they are utilised for breeding or food. Females of several species prefer low frequency calls as an indication of a male's larger size and thus potential fitness (Morris & Yoon, 1989; Ryan, 1980; Wilbur et al., 1978). This preference for larger males would suggest that females can detect the presence of smaller males in the environment that are less suitable for mating but easier to prey upon, from a distance based simply on their call. Indeed, amphibian females may consider only a small proportion of a breeding pool of males to be suitable to mate with (O'Brien et al., 2018), with a large proportion of males instead considered potential food items. In other animals, smaller males have a higher probability of being cannibalised than larger males (e.g. Elgar & Jones, 2008). As amphibians have indeterminate growth, this could mean that younger and thus smaller males that have just become reproductively active could face more extreme predation pressure from adult females.

It is expected that the hormonal response of females towards males during the breeding period is to exploit them as a mate (Shankar & Whitaker, 2013); akin to the response of females to their offspring during the breeding period when predatory behaviours are suppressed (Waldman, 1991). Our observation occurred in the middle of the *L. aurea* breeding season but during a period of low activity caused by dry conditions and low rainfall that likely resulted in temporarily suboptimal conditions, which may have triggered a shift in resource priorities for females towards nutrient acquisition and away from any interest in reproducing. What this could show is that the relative value of adult males as a potential mate versus food resource may vary over time and between individuals, with higher quality mates less likely to be exploited as a food source during active breeding episodes. In species where sexual cannibalism is common, females could be acting as a selecting force on males by mating with those of higher quality and removing those of poorer quality via cannibalism; although we do not suggest this is occurring in *L. aurea* specifically.

Our study provides an initial exploratory evaluation into the presence of sexual cannibalism among amphibians, indicating that the high incidence of sexual dimorphism within this group may afford females an ability to exploit their size advantage for predation on males. It is possible that our results are currently biased by the limited number of records of adult‐adult predation in this group and can only be confirmed with larger sample sizes in the future. Additionally, several of our records are based on captive interactions between individuals, where confinement under laboratory conditions may promote abnormal cannibalistic behaviours. We thus encourage authors to publish observations of cannibalism, including unsuccessful attempts, under natural conditions when possible. Despite these limitations, our preliminary findings contribute to the broader understanding of cannibalistic behaviours in amphibians and underscore the potential significance of sexual cannibalism in this group. Further research is warranted to ascertain the frequency of its occurrence in amphibians and its implications for population dynamics during breeding periods when adults are more likely to be aggregated.

## AUTHOR CONTRIBUTIONS

**John Gould:** Conceptualization (lead); data curation (lead); formal analysis (equal); investigation (equal); methodology (equal); visualization (lead); writing – original draft (lead); writing – review and editing (equal). **Chad T. Beranek:** Formal analysis (equal); investigation (equal); methodology (equal); writing – original draft (supporting); writing – review and editing (equal).

## CONFLICT OF INTEREST STATEMENT

The authors declare no conflicts of interest.

## Supporting information


Data S1

## Data Availability

Data have been provided as Data‐S1.
